# The prevalence of chronic pain in orchestra musicians

**DOI:** 10.3205/000242

**Published:** 2017-01-12

**Authors:** Elena R. Gasenzer, Marie-Juliana Klumpp, Dawid Pieper, Edmund A. M. Neugebauer

**Affiliations:** 1 Institute for Research in Operative Medicine, Witten/Herdecke University, Campus Cologne-Merheim, Cologne, Germany; 2 Medical School Brandenburg Theodor Fontane, Faculty of Health, Neuruppin, Germany; 3 Interdisciplinary Centre for Health Services Research, Witten/Herdecke University, Witten, Germany

**Keywords:** chronic pain, musician, orchestra, music instruments, prevalence

## Abstract

**Background:** The study investigated the incidence of chronic pain as well as causes and mechanisms of pain chronification in orchestra musicians.

**Aims:** Chronic pain is a serious problem in the study group due to very specific playing techniques and body positions while playing, with a high impact on professional and private life.

**Methods:** 8,645 professional musicians from 132 German cultural orchestras were contacted and asked about chronic pain via an online questionnaire. The study group comprised orchestra musicians suffering from pain. The control group consisted of musicians playing the same type of instruments (same working conditions) who reported to be free of pain.

**Results:** The response rate was 8.6% (740 musicians). 66.2% (n=490) out of 740 musicians who completed the questionnaire reported chronic pain. The most frequently reported localizations of pain were the body parts which are mostly involved in instrumental playing such as back (70%), shoulders (67.8%), neck (64.1%), hands and wrists (39.8%). 27.4% of the investigated musicians suffered from pain with a high degree of impairment.

**Conclusions:** These results appear conclusive and indicate a need to continue research into chronic pain in musicians.

## Zusammenfassung

**Hintergrund:** Die Studie beschäftigte sich mit dem Auftreten chronischer Schmerzen, ihren Ursachen und ihren Mechanismen bei (klassischen) Orchestermusikern.

**Ziele:** Chronischer Schmerz ist ein weitreichendes Problem unter Orchestermusikern. Ursachen sind die sehr spezielle Haltung und Spieltechnik der verschiedenen klassischen Orchesterinstrumente, sowie lange Spiel- und Übezeiten. Der chronische Schmerz des Orchestermusikers hat weitreichende Auswirkungen auf seine künstlerische Tätigkeit wie auch auf alltagspraktische Aktivitäten.

**Methoden:** Es wurden 8.645 professionelle Musiker deutscher, öffentlich geförderter Sinfonie-, Opern-, Konzert- und Rundfunkorchester kontaktiert und mittels eines Online-Fragebogens zu ihren akuten oder chronischen Schmerzen befragt. Die Kontrollgruppe bildeten Musiker aus denselben Orchestern, die angaben, keine Schmerzen zu haben.

**Ergebnisse:** Die Rückantwortquote betrug 8,6% (n=740). 66,2% (n=490) der Befragten gaben an, unter Schmerzen zu leiden. Bezüglich der Lokalisation wurden Körperpartien genannt, die beim Spiel der verschiedenen Instrumente am stärksten belastet werden, wie Rücken (70%), Schultern (67,8%), Nacken (64,1%) sowie Hände/Handgelenke (39,8%). 27,4% der Teilnehmer gaben an unter Schmerzen zu leiden, die sie insgesamt sehr stark beeinträchtigen.

**Schlussfolgerung:** Die Ergebnisse der Studie zeigen, dass chronische Schmerzen bei Orchestermusikern ein häufiges und sehr ernstes Problem sind, dessen Mechanismen und Ursachen umfassend erforscht werden sollten.

## Introduction

Several studies indicate an increasing number of musicians with occupational disorders and a need for treatment and consultation [1], [2], [3], [4]. Unlike musculoskeletal disorders [5], [6] or neuromuscular dysfunctions [7], [8], chronic pain has yet received little attention in musician’s medicine [9]. Occupational illnesses among musicians still pose a potential threat to a successful career, with the danger of chronic pain as a particular problem [6].

The focus in musician’s medicine is on musculoskeletal disorders, providing a clear picture of which instrument-specific symptoms occur under which circumstances [6], [10], [11], [12]. High string players typically also suffer from overuse of jaw, chin and neck, due to the strain of holding the instrument with chin and shoulder [1], [2], [13], [14], [15]. Injuries, overuse, and dysfunctions of hands are mainly in the focus of musician’s medicine [16]. The emphasis here is on tendosynovitis and carpal tunnel syndrome due to overuse [17], [14]. Neurological complaints in musicians such as focal dystonia [2], [13], [18] provide a broad field for research. Various studies see the neurophysiological cause of movement disorder in basal ganglia [18] or the thalamus [19] and in an anomaly of the sensorimotor organization of the cortical area associated with the affected hand [8]. Since focal dystonia constitutes an acquired faulty programming of the brain [20], [21], there is a clear parallel between the dysfunction of neuronal programming and the chronification of pain [22].

This research project addresses, among other issues, the specific causes leading to chronic pain in affected musicians. We describe in particular those results that refer to the frequency and intensity of chronic pain in members of publicly funded German symphony, radio, theatre, and opera orchestras.

## Methods

The design is a cross-sectional study in which the study group comprised orchestra musicians suffering from pain. The control group consisted of musicians playing the same type of instruments who reported to be free of pain. The period of recruitment in which the online questionnaire (Attachment 1) was available covered 14 weeks in summer 2013. The Ethics Committee of Witten/Herdecke University gave their statement on ethical approval (No. 43/2013).

The selection of orchestras and of the study group was restricted to approximately 130 publicly funded classical symphony, chamber, and radio orchestras in Germany. These orchestras altogether employ a total of 8,645 musicians; so this was the number of potential study participants nationwide. As a first step, all professional symphony, chamber, and radio orchestras that receive public funding in Germany were contacted with the request to participate in the study. The covering letter was mailed to the orchestra boards, who forwarded it to orchestra members via internal emailing lists. A written letter that contained a description of the method, the goal of the study, and facts about data privacy protection was sent to the participants to draw the interest of the musicians. The aim of this broad information and the possibility for the participants to contact the study group via email, fax or phone was to increase the participation of the musicians. Three musicians contacted us by mail in order to send some literature about the topic or made suggestions regarding the layout of the questionnaire.

The aim of the survey was to identify musicians who suffer from chronic pain, and to make assessments on possible causes of the chronification of pain in orchestra musicians.

Interventions refer to the various musical instruments used in classical orchestras (violin, viola, violoncello, double bass, flute, oboe, clarinet, bassoon, trumpet, trombone, horn, tuba, percussion instruments, harp). Interventions also refer to professional experience, entrance age, and mental disposition. Possible responses to the question about incidence of pain are “yes” and “no”, and the pain scale designed by von Korff and used in this questionnaire offers a gradation in four degrees of chronic pain.

The questionnaire (Attachment 1) addressed in particular the specific activities of musicians, such as practicing habits and occupational strains. In order to permit precise and considered answers to questions about incidence, location, type and intensity of pain, as well as general questions about physical well-being, the online questionnaire was based on the validated and internationally recognized pain questionnaire (DSF) issued by the *German Association for the Study of Pain* [23], [24], [25]. It is important to note that the von Korff scale does **not** measure the **intensity of pain**, but the **extent of disability caused by pain**. With the aim in mind to get comprehensive results from measuring the intensity of pain as well as the degree of impairment in various activities caused by pain, the authors used numbered rating scales comparable to the von Korff scale. 

The software IBM SPSS Statistics 21 was used for data evaluation and statistical analysis. Relations between variables were evaluated via cross tabulation with chi-square test. An explorative data analysis was performed by t-test to investigate differences among groups. The method chosen to investigate differences among groups was univariate variance analysis. The target variable was chronic pain (momentary or recurring) associated with a grade of intensity of 1 or higher on the von Korff scale. We distinguished between three groups: no pain group, low pain group, and high pain group. Measurable statistical variables were examined for correlations. The goal was to identify causal variables of the causes of chronic pain in professional musicians. Noticeable variables were examined by multivariate analysis.

## Results

**Figure 1:**
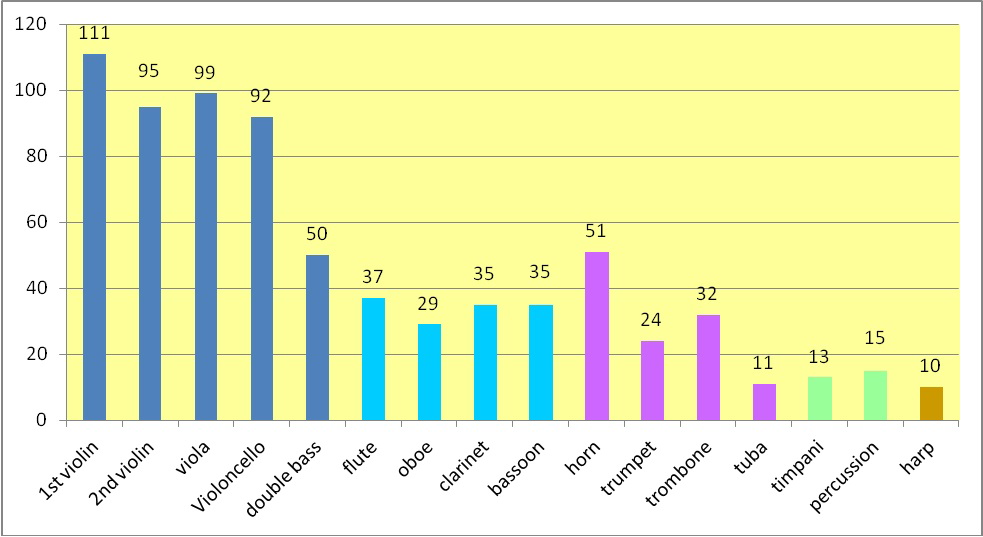
Distribution and frequency of instruments among respondents (total: n=740)

The potential study group comprised 8,645 musicians employed in symphony, radio, and opera orchestras that receive public funding in Germany. A total of 740 duly completed questionnaires (8.6%) was returned and served as a basis for evaluation (Figure 1 (Fig. 1)). 42.3% (n=313) of musicians were female, 57.7% (n=427) were male. The average age of musicians (question 3) was 46.4 years, with a standard deviation of 9.5 years. The youngest respondents were 22, and the oldest 65 years of age.

The average scope of professional experience as an orchestra musician was 23.3 years with a standard deviation of 10.3 years. Pertinent figures varied between 3 and 49 years. Most respondents have played in several orchestras over the course of their professional lives. At the time of the survey, study participants had been playing for 18.9 years on average in the orchestra where they were permanently employed during the investigation period, with a standard deviation of 10.2 years. The highest figure given here was 43 years. 182 out of 740 respondents reported that they moved to another city more than once due to a change of employment.

**Figure 2:**
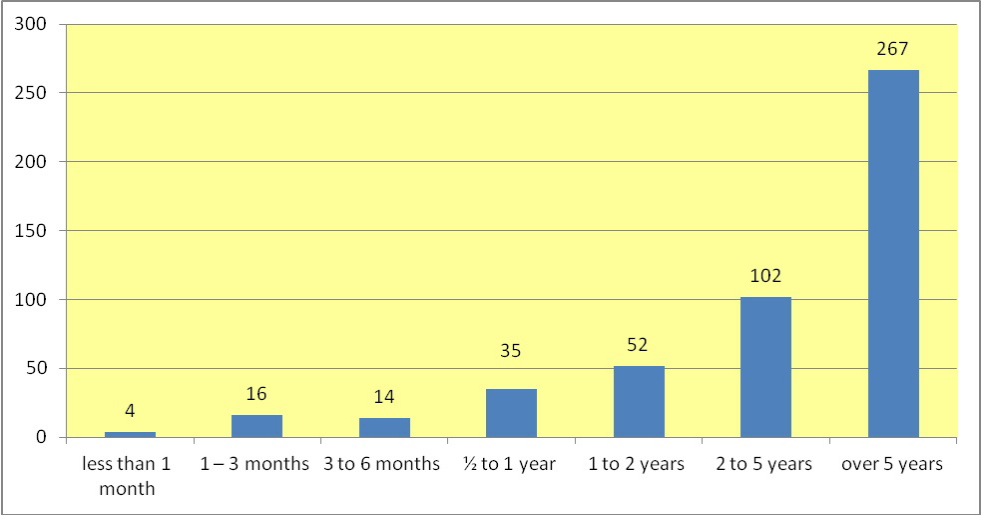
Duration of pain in number of musicians (n=490)

66% (n=490) of 740 participants reported current or recurring pain. In this study pain was defined as “chronic” if it occurs repeatedly or continuously over a period of more than 3 months. Out of n=490 musicians who reported pain, n=470 (63.5% of all respondents) suffered from continuous pain for more than 3 months (Figure 2 (Fig. 2)).

**Figure 3:**
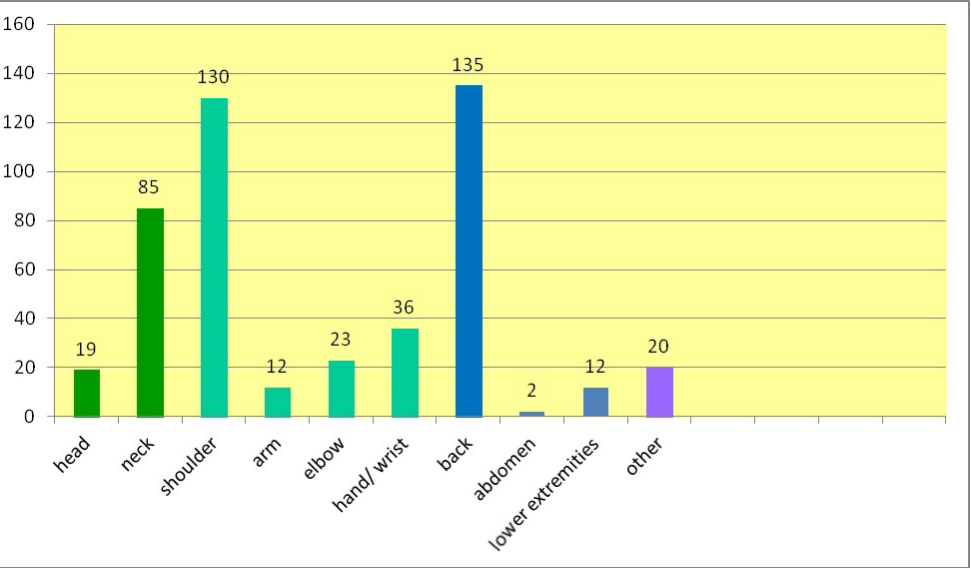
Localization of pain musicians suffered most (10 locations were mentioned; “lower extremities” contained buttocks, knee, angle joint, feet; n=490)

A clear picture emerged from detailed information for localization of pain, reflecting the distribution of instruments in the orchestra and the specific body posture required to play them. Musicians mainly indicated pain in the upper body and upper extremities. Back pain in particular was reported most frequently by n=135 musicians (Figure 3 (Fig. 3)). 

The different groups of instruments provided a clear picture of the typical locations of pain. These results showed correlations between instrument and pain location. The high strings were the group with the highest rate of chronic pain in the shoulder (1^st^ violin n=62, 2^nd^ violin n=18).

Back pain was another symptom typical of high string players (1^st^ and 2^nd^ violin n=40, viola n=19). Players of wind instruments show typical locations of pain as well. The shoulder is the most vulnerable body part, especially in high strings and flute players. The reason for this is the characteristic body posture and the playing technique. In case of high string instruments, the left arm and shoulder remain in an abduction position with an elevation of the forearm and hand when playing. The shoulder joint and the muscles of the rotator cuff are extremely stretched, whereas the shoulder and elbow of the right bow arm are very stressed by specific styles of bow technique, which are necessary in different expressive styles like *détaché*, *staccato*, *spiccatto* and *col legno*. In case of these string techniques the movement of the bow arm will suddenly be stopped or the bow will hit the string and bounce back due to the tension of the string, of the wooden bar of the bow, and of the bow hairs. This mechanical power stresses the muscles, tendons, and structures of the right shoulder and elbow extremely. 

In the wood wind players back pain was the most quoted location (flute n=8, clarinet n=5, oboe n=5, bassoon n=5). The shoulder was also mentioned as a much affected body part (n=13), followed by pain in hands and wrists. 

The back was the most frequently reported pain location in brass players (horn n=9, trumpet n=1, trombone n=9, tuba n=4). The example of back pain shows that size and weight of the instrument, especially wood wind and brass instruments, play an important part in the development of chronic pain.

**Figure 4:**
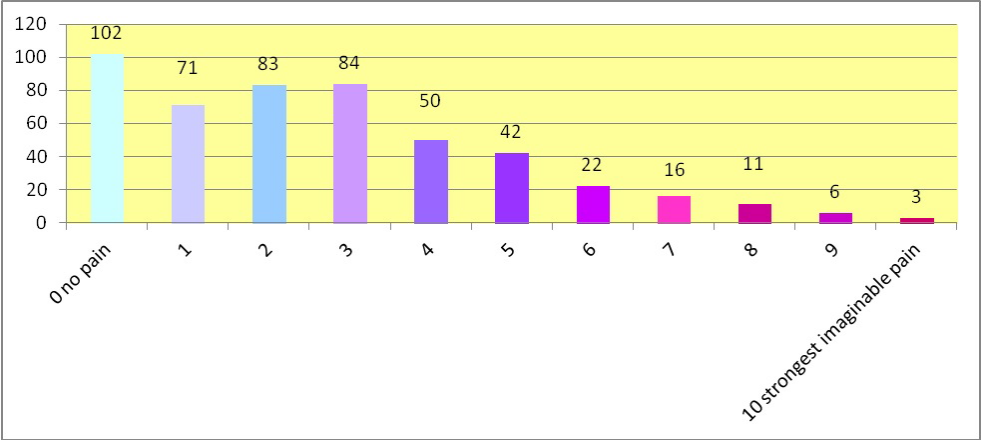
Actual pain: pain intensity at the time of completing the questionnaire (number of musicians; total: n=490)

Question 19 addressed the extent of impairment due to pain. In particular, study participants were requested to indicate and estimate the level of pain. The respective questions contained a scale from 0 (no pain) to 10 (strongest imaginable pain). The first question asked for the level of current pain (i.e. while completing the questionnaire). The mean level of pain indicated here was 2.7 (standard deviation 2.3). A total of n=234 musicians reported a current pain level of 3 and more. N=102 musicians reported no pain while completing the questionnaire (Figure 4 (Fig. 4)).

**Figure 5:**
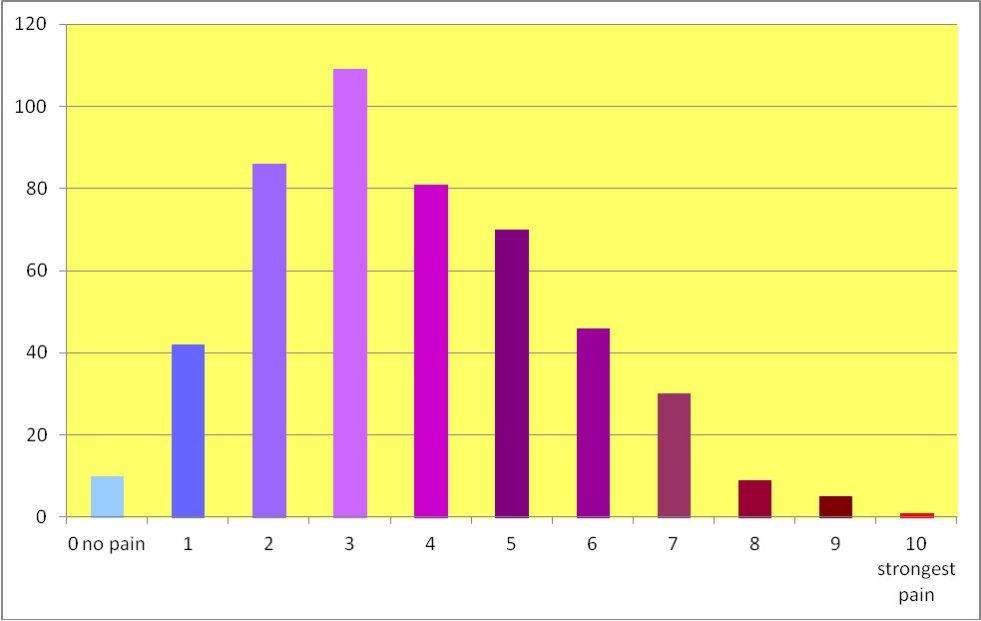
Pain intensity on average of over the past four weeks in number of musicians (n=490)

**Figure 6:**
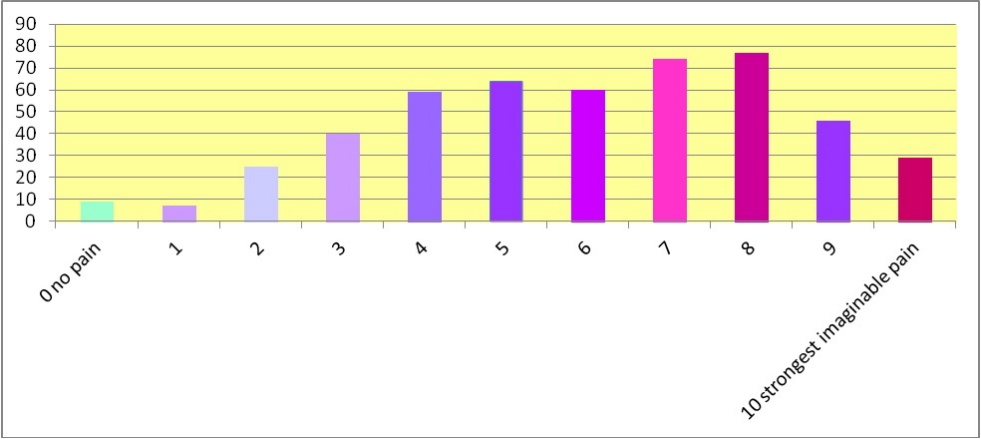
Maximal pain felt within the last four weeks in number of musicians (n=490)

The averaged pain intensity within the past four weeks prior to the survey reached a mean value of 3.8 with a standard deviation of 1.9. N=352 musicians indicated that they suffered pain at a level of 3 and more over the past four weeks (Figure 5 (Fig. 5)). The maximum pain intensity was given with a mean value of 6.0 with a standard deviation of 2.4. N=449 musicians indicated a value of 3 and higher (Figure 6 (Fig. 6)).

89% (n=440) in the group of musicians with pain attributed their pain to a specific form of physical stress. Only 9% (n=44) believed the cause to be a specific illness.

49% (n=242) in the group of musicians with pain (n=490) aim to influence their pain experience proactively by doing sports and soft sports. The best way to reduce pain is for 17% (n=84) to undergo special (medical) treatment; 11.4% (n=56) use medication, 7.1% (n=35) use relaxation techniques, 6% (n=32) try to avoid the pain, 4% (n=20) believe in taking a rest and 3% (n=15) prefer to ignore the pain. 1.2% (n=6) either have no strategy to influence their pain proactively, or their strategy is to do nothing about it.

45% (n=221) in the group of musicians with pain suffered from additional chronic diseases such as cardiovascular or neurological disorders, compared to only 23% (n=67) in the group of musicians without pain. N=333 of participating musicians attributed their pain directly to their instrumental play, e.g. to the weight of the instrument, the required body position, and not least the duration of daily exercises, including overly long and frequent performances and rehearsals, monotonous posture, and one-sided movements.

Respondents’ self-rated health status, however, contradicts the reported incidence of pain. 5% (n=26) in the group of musicians with pain described their state of health as excellent, 29% (n=145) as very good, 50% (n=246) as good, 12% (n=62) as not so good and only 2% (n=11) as poor. In the group of musicians without pain, the general health status was assessed as better: 11% (n=28) rated their health as excellent, 45% (n=113) as very good, 38% (n=97) as good, 4% (n=12) as not so good. In this group, no one described his health status as poor. Contradictions were found in ratings given for the incidence of pain – with the patients’ individual perception as the only measuring instrument – and respondents’ self-reported health status. The status of health was addressed in a 5-point scale with verbal descriptions. The questionnaire was complemented by an “artist questionnaire” inquiring about main musical activities to reveal possible correlations between a musician’s chronic pains and his or her major artistic focus. 

Only a minority of musicians availed themselves of specialist medical care. A number of outpatient wards in Germany offer consultancy services specifically geared to the needs of musicians. 42% of interviewees (n=316) reported that they know about these facilities. The percentage of those who consulted specialist services in musician’s medicine was only 13% (n=66).

21% of respondents (n=156) have already consulted such a ward, 63% (n=99) because of pain, 7% (n=57) for other reasons.

Over 50% of musicians in the study group reported permanent pain. The severity of chronic pain was measured on the scale designed by von Korff [23] which connects the severity of pain with the degree of impairment. Most musicians (n=284, 38%) reported permanent slight pain (Grade I) with an impairment of 3 disability points. N=104 (14%) persons reported a high intensity of pain (Grade II) with an impairment of 3 disability points. N=60 (8%) musicians suffered from strong pain (Grade III) with a high degree of impairment of 4 disability points. N=39 (5%) musicians reported very strong pain (Grade IV) with a very high degree of impairment of 5–6 disability points.

## Discussion

The participant rate of 8.6% in our study was not high enough to represent a sizeable population. Such studies normally have response rates of one third to 50% [26], [27], [28]. High response rates reach nearly 70% [29].

We saw the reason for this low response rate in the special situation of employed orchestra musicians in their typical manner to accept health problems. In Germany, employed musicians of public symphony, theatre, and opera orchestras are employed as public servants. Employments in such state institutions are rare and treasured [30]. Because of this difficult situation, only a few musicians are willing to provide information about their health status or diseases [31], [32], [33]. We perceive these facts as the reason for the small response rate of 8.6%. 

In our opinion, strengths and weaknesses of the study can be meaningful. The small response rate shows that many musicians are afraid that their health problems will become public. Due to this problem, a response rate of 8.6% is representative for the total group of musicians. The low response rate shows at least the problem of the acceptance of pain and health problems in German orchestras, as well as the occurrence of such problems. This should be a cause to develop management strategies for the treatment of chronic pain.

The high number of musicians with strong and chronic pain grade III and IV [23] in the study group indicates the need for further research and development of effective treatment concepts in the field. 66% (n=490) out of 740 participants reported current or recurrent pain. These results resemble those from a recent systematic review of 30 included studies which investigated pain in musicians [34]. The prevalence of pain in the investigated studies was between 29% and 88.6%. Studies asked for point prevalence (weighted mean prevalence 64.9%), 1-week prevalence (up to 74.3%), 1-month prevalence (56–71%) and 1-year prevalence (ranging from 29% to 88.6%). 

In accordance with the distribution of orchestra instruments, string players formed the largest group of participants. High string players (1^st^ and 2^nd^ violin n=40, viola n=19) were the group with the highest rate of chronic pain in shoulder and back. The back was also the location of chronic pain most frequently reported by wood wind and brass players, percussionists, and harp players. These results show that the body parts of back and shoulder are a much neglected field in the physical part of musical education. The classical methods of instruction in orchestra instruments were established in the 19^th^ and early 20^th^ centuries. The goal is the perfect mastery of the instrument, whereas the musician’s body is of no relevance. 

The complex problems involved in and the causes of chronic pain were well known in the group of participants suffering chronic pain, and most musicians tried to address the problem in a proactive manner. Only a small group used medication. Others engaged in some soft sports or fitness activities to reduce pain, underwent special treatment or performed relaxation techniques. Only 3% ignored the pain. 1.2% reported that they have no strategy to influence their pain proactively, or their strategy is to do nothing about it. 

In view of these results, it is surprising to note that most musicians who suffered pain did not equate pain with illness. A lot of musicians with pain described their state of health as excellent, very good or good. This fact suggests a considerable problem and inconsistence in the treatment and diagnosis of musician’s maladies: the rate and the intensity of playing related to chronic pain may be very high but in the perception chronic pain is not necessarily of pathological significance. This is the core problem in the research, treatment, and evaluation of chronic pain in musicians.

The reported lifetime prevalence varied between 58.7% and 81%. A study with 720 professional musicians from 10 classical orchestras in Berlin, Saxony and Saxony-Anhalt [13] reported a rate of current or past playing-related musculoskeletal pain in 89.5% of participants. 62.7% reported pain during the last 3 months whereas 8.6% suffered from permanent pain. These results confirm the findings of our study and underline an urgent need for extensive research of chronic pain syndromes in musicians. 

This investigation was the first to include nearly all publicly funded classical orchestras in Germany. The study was the first to examine the prevalence and intensity of pain in relation to the severity of impairment to mental state, physical health, and quality of life. Findings showed that chronic pain in orchestra musicians caused by the playing of instruments is a widespread problem with a strong impact not only on the affected musicians’ quality of life but also on cultural life in general. The most surprising survey result was that the investigated musicians generally reported a good state of health, irrespective of reported high levels of pain and impairment. Other investigations of classical orchestras did not report corresponding findings. 

There is a general consensus among experts in pain medicine that pain at a level of 3 and higher constitutes a serious impairment to quality of life and the ability to cope with daily activities [19], [23]. A total of 234 musicians reported pain levels of 3 and higher for the past four weeks. 449 musicians reported a level of 3 and higher for the pain perceived as worst within the past four weeks. This high number of musicians who reported permanent pain with a high level of impairment indicates the necessity of professional medical treatment and of rehabilitation programs for musicians with chronic pain syndromes. 

The locations of chronic pain indicated by musicians clearly reflect the specific postures and types of stress associated with playing different types of orchestra instruments. Shoulder and neck were most frequently affected in the area of upper extremities. For most instruments (high strings, wood, and brass players), muscles in throat and neck, shoulders and upper arm assume the supporting work required to maintain the instrument in the specific position for play [10], [11], [12], [34], [35]. This corresponds to the experience won in musician’s medicine that those extremities which are mainly involved in playing an instrument are most frequently affected by syndromes of overstress, pain or inflammatory processes caused by overuse [36].

Pains in the upper extremities, arms, elbows, wrists, hands/fingers were reported in second place – an expected result since these body parts perform the actual work in playing an instrument. Syndromes of overuse are exclusively due to the extremely high number of continuously repeated movements. While the supporting and postural musculature may easily be trained and strengthened via physiotherapy or movement training, these interventions are almost impossible to apply to distal upper extremities. Responses with regard to possible causes of chronic pain reveal that most musicians are aware of the problems of pain. The supporting apparatus in the areas of back, shoulder, and neck mainly assumes the static strain, whereby size and weight of some instruments may be considerable. In addition, these body parts are moved little in musical play, with extreme tension as consequence and an acquired elevated muscle tone.

The back was the most frequently named body part in string players, wood wind and brass players. The majority of musicians saw playing their instrument as a cause of the pain. A maximum of 8 hours per day for musical exercises in the sitting position was reported. Some instruments like violoncello or harp played exclusively in the sitting position must be noted in this context. Today the exposure time through practicing and instrumental playing as well as the amount of performances under psychological stress are assumed to be the most important risk factors for the development of musician’s maladies and pain syndromes [36]. The distribution of indicated body areas shows that mainly those body parts which are immediately involved in supporting the posture and in playing the instrument are affected by chronic pain.

These results are alarming in view of the level of impairment and loss of quality of life which musicians suffer as a consequence of chronic pain. They indicate an urgent need for information and sensitization. Musicians must be motivated to address their health problems openly and consult specialists at an early time. Frequently, chronic pain develops precisely because the problem is ignored and no effective pain therapy is applied during the acute stage. Affected persons often continue their normal activities despite injuries or overuse syndromes over long periods, or try to cope with health problems unassisted [37]. This behavior reduces the chances of effective pain therapy and professional rehabilitation. Information is particularly important in this respect so that pain therapy can start early and chronification of pain can be prevented. 

42.7% (n=316) of musicians in our study group were informed about outpatient facilities for performing arts medicine or knew about specialist physicians, ambulances, and clinical centers. 57.3% (n=424) had no knowledge of such facilities. Only 13.4% (n=99) actually visited a center of this type because of their pain. 

## Conclusions

We investigated musicians employed with orchestras in the Federal Republic of Germany. The study can only reflect conditions in Germany. Results of international studies in this field are similar, but conditions are always different and highly individual for the investigated groups. There are differences in the starting age of musical education and the methods employed between countries in Europe, Asia, and America, just as styles of playing and repertoires are different. 

The number of respondents, i.e. 740, indicates a great deal of interest among study participants in health-related issues. The demand for information and assistance with regard to the chronification of pain caused by musical activities is substantial. Compared to orthopedic, sports-medicine related or neurological diseases and disorders, chronic pain receives too little attention in musician’s medicine, among musicians themselves, in orchestras, and in research. Data won from the questionnaire suggest that musicians still tend to put up with chronic pain, rather than undergo treatment in a therapy facility specialized in musician’s medicine. These differences and characteristics must be considered in planning such studies. 

It is therefore desirable to improve networking between orchestras and outpatient wards for musician’s medicine, as well as communication between musician, pain therapist, attending physician, and physiotherapist. This will also require orchestra boards and managers to address the issue. The aim must be to develop concepts for health promotion in the planning of orchestra services and performance schedules, in collaboration with musicians and outpatient wards specialized in musician’s medicine [38], [39]. The time has come for comprehensive revisions in classical programs of instrumental training. Instrumental education must include due consideration of the body movements involved in playing and their implications for health. A number of music academies and conservatories in Germany have established outpatient services and offer specific classes in performing arts medicine, anatomy and physiology. Many musicians react only when it is too late, when the process of developing an overuse syndrome or chronic pain has already started. Prevention must start earlier. Faulty instrumental techniques and body movements, which cause music-related illnesses start impairing instrumental play at an early stage in music education. We are in the process of establishing an efficient health care delivery system for musicians. Now the music academies will be called upon to educate students – and instructors, those of children in particular – how to make music and stay healthy. 

## Notes

### Competing interests

The authors declare that they have no competing interests. 

Data contained herein were published in the context of the doctoral thesis (Faculty of Health, School of Medicine) of M.J. Klumpp.

## Supplementary Material

Questionnaire: Chronic pain in orchestra musicians
