# Supplementary material for: The prevalence of chronic pain in orchestra musicians
Source: Ger Med Sci. 2017 Jan 12;15:Doc01. doi: 10.3205/000242 (PMC5238713; doi:10.3205/000242)
Supplement: Questionnaire: Chronic pain in orchestra musicians [file GMS-15-01-s-001.pdf]

Dear study participant,

chronic pain constitutes a considerable health risk for **professional musicians** and a potential threat to a successful career.

The purpose of our research project is to explore the specific individual processes towards, and causes of, chronic pain in musicians. Another objective is to develop strategies and effective methods to treat chronic pain.

You can give us valuable help!

Please complete the questionnaire below.

This will only take approx. 15 minutes of your time!

Is the survey anonymus?
Yes, the survey is anonymous. Only anonymized data are collected without any direct reference to your person. None of the information will be disclosed to a third party. Data will be evaluated anonymously and destroyed upon conclusion of the project. Evaluated study results will be published in a journal without any conclusions to be drawn about individual participants.

Is the participation voluntary?
Participation is voluntary, of course. You may withdraw your consent at any time without stating reasons. Participation will in no way prevent a continuation of treatment and medication prescribed for existing complaints. The Ethics Committee of Witten/Herdecke University has given approval of the survey. Parts of our questionnaire are based on the official Deutscher Schmerzfragebogen, Deutsche Schmerzgesellschaft e.V., Version 2012.2, SF-36 Hogrefe Verlag, Bullinger u. Kirchberger 1998 and Audit-C.

Please feel free to contact us at any time with any questions you might have.
Please start now with the questionnaire.
Thank you!

Univ.-Prof.Dr.Prof.h.c.E.Neugebauer
Leitung des Instituts für Forschung in der Operativen Medizin, Köln-Merheim

Cand.med.M.-J.Klumpp
Studentin der Humanmedizin Universität Witten/Herdecke

Dr.rer.med.E.R.Gasenzer
Institut für Forschung in der Operativen Medizin, Köln-Merheim

IFOM - Institut für Forschung in der Operativen Medizin, Direktor: Univ. - Prof. Dr. Prof. h.c. Edmund A. M. Neugebauer, Lehrstuhl für Chirurgische Forschung, Fakultät für Gesundheit, Department für Humanmedizin, Universität Witten/Herdecke Ostmerheimer Str. 200, Haus 38, 51109 Köln, Telefon +49 221 98957-0, Fax +49 221 98957-30
E-Mail: ifom-neugebauer-sek@uni-wh.de

General questions

1. In which type of orchestra you are playing? (theater orchestra or oper orchestra, concert orchestra, chamber orchestra, radio orchestra, other)

____

2.Gender: O female O male

3.Age (in years): ________

Fragen zum Instrumentenspiel Teil 1

4. Please indicate the chief instrument you play in your orchestra:

O 1st violin O 2nd violin O bassoon O flute O harp

O horn O clarinett O double bass O oboe O percussion

O timpani O trombone O trumpet O tuba O viola O violoncello

O other: ______________

5. For how many hours per day do you play your chief instrument?

Daily duration of playing: ________ hours

... hours standing: ________ hours

... hours sitting: ________ hours

6. When did you start getting instruction in your chief instrument?

In the age of ________ years

7. Which technical equipment do you use in playing your chief instrument?
Please choose all that apply:

O no technical equipment O chin / shoulder support O straps O grips

O chair cushion O footrest O foot end caps

O lighting O cold protection O hearing protection

O other: _____________________

Fragen zum Instrumentenspiel Teil 2

8. Frequency of performance?

Number of concerts last week ___________

Number of concerts last month ___________

Number of concerts last year ___________

9. Of the number of concerts last year I played...

… in the orchestra ________ concerts

... in a small ensemble ________ concerts

... as a solist ________ concerts

10. I have been active in this orchestra for about...

... years __________

and ... months __________

11. I am still in my probationary period in the current orchestra

O yes O no

12. I have former work experience in orchestras of altogether...

... years __________

13. I have often had to move to another city to pursue my musical activities

O yes O no

13a. If so, how often? _______ times

Questions related to pain

14. Are you suffering from pain (in this moment, or from recurring pain)?

O yes O no (You will continue with question 24)

15. Look at these figures::

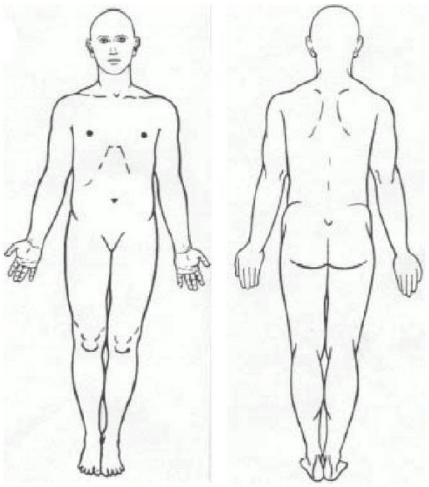

Bildquelle: DSF, Frage 5, S.3

Indicate the body region where you feel pain.

<u>Tick off</u> one or several <u>answers</u>.

O head O neck O shoulder O elbow O wrist and hand

O upper back O lower back O buttock O chest O abdomen

O hip O knee O ankle joint and foot

O other body regions: ____________________

15a. Please also indicate the affected side of the body where you feel the pain:
left or right or both sides.
("Both sides" can also mean "sometimes the one side, somtimes the other side")

head:	O left	O right	O both sides
neck:	O left	O right	O both sides
shoulder:	O left	O right	O both sides
elbow:	O left	O right	O both sides
wrist and hand:	O left	O right	O both sides
upper back:	O left	O right	O both sides
lower back:	O left	O right	O both sides
buttock:	O left	O right	O both sides
chest:	O left	O right	O both sides
abdomen:	O left	O right	O both sides
hip:	O left	O right	O both sides
knee:	O left	O right	O both sides
ankle joint and foot:	O left	O right	O both sides
Other body regions: ____________________			

16. Which of the indicated pains are <u>most severe?</u>

____

17. How long have you been feeling this pain?
Only answer this question if the following conditions are met:

O less than 1 month O 1 to 3 months O 3 to 6 months

O 1/2 to 1 year O 1 to 2 years O 2-5 years O more than 5 years

Nach: DSF, Frage 7a, S.3

18. Which of the statements below describes best your pains over the past four weeks?

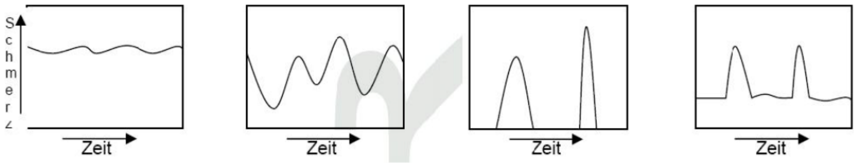

Only answer this question if the following conditions are met:

O background pain with slight variations

O background pain with strong variations

O pain attacks, no pain in between

O pain attacks with background pain in between

DSF, Frage 8a, S.4

18a. If you suffer from pain attacks, how often do they occur on average?

O several times a day O once per day O several times per week

O once per week O once per month O less frequently:___________

DSF, Frage 8b, S.4

18b. Average duration of these attacks?

O seconds O minutes O hours

O up to 3 days O more than 3 days

DSF, Frage 8c, S.4

Intensity of pain

19. Describe the intensity of pain. Tick off the pain level on a scale from 0 to 10.

For your orientation: 0 means no pain, 10 means you suffer the worst imagineable pain.

First, please indicate your <u>current level of pain</u>:

	no pain									strongest pain
current level of pain	1	2	3	4	5	6	7	8	9	10

DSF, Frage 11.a, S.5

Now please indicate the average pain level over the past 4 weeks:

	no pain									strongest pain
average level of pain	1	2	3	4	5	6	7	8	9	10

DSF, Frage 11.b, S.5

Now please indicate the highest level of pain experienced during the past 4 weeks:

	no pain									strongest pain
highest level of pain	1	2	3	4	5	6	7	8	9	10

DSF, Frage 11.c, S.5

Impact of pain

20. The following questions address your pain over the past 3 months. We want more details on the impact of your pain during this period.

On how many days in the past 3 months did your pain prevent you from performing your habitual activities (job, leisure time, household chores)?

On about __________ days

DSF, Frage 12.a, S.5

To which extent did pain affect your daily life over the past 3 months (dressing, washing yourself, eating, shopping etc.)? Tick off the relevant figure on the scale below:

	no impact									total impact
Impairment in daily life	1	2	3	4	5	6	7	8	9	10

DSF, Frage 12.b, S.5

To which extent did pain affect your activities in leisure time, with family or friends over the past 3 months? Tick off the relevant figure on the scale below:

	no impact									total impact
Impairment in leisure activities	1	2	3	4	5	6	7	8	9	10

DSF, Frage 12.c, S.5

To which extent did pain affect your working capacity (including household chores) over the past 3 months? Tick off the relevant figure on the scale below:

	no impact									total impact
Impairment of working capacity	1	2	3	4	5	6	7	8	9	10

DSF, Frage 12.d, S.5

Coping with pain

21. Does the pain occur while you play the instrument?

O always O mostly O often O sometimes O rarely O never

21a. Does the pain occur only while you play the instrument?

O yes O no

22. What do you do to positively influence your pain?
Please give exact details, such as going for a walk, sleeping, distraction, use of medication etc.

Rate according to significance, i.e. mention first what helps best:

____

Nach: DSF, Frage 14, S.6

23. To which causes do you attribute your pain? (Multiple answers possible)
Please choose all that apply:

O no cause I can identify

O some specific disease — which specific disease exactly? ____________

O physical stress — which physical stress exactly? ____________

O emotional stress — which emotional stress exactly? ____________

O a different cause — which different cause exactly? ____________

Nach: DSF, Frage 13, S.6

Health and well-being

24. How would you describe your general state of health?

O excellent O very good O good O not so good O bad

DSF, Frage L-1, Seite 13, SF-12 Hogrefe Verlag, SF-12 Fragebogen von Bullinger und Kirchberger

25. I engage in physical exercise or relaxation techniques for at least 30 minutes
such as yoga, muscle relaxation according to Jacobsen, jogging, weight training, gym, tai chi, autogenic training, other

O no per week O 1x per week O 2-3x per week O more than 3x per week

25a. Which type of sports or relaxation techniques exactly?

____

26a. Please assess your current state of well-being. Please indicate how you felt most of the time during the past 14 days. Tick off the most accurate figure on the 6-step scale: 0 = not correct at all, 5 = fully correct. Please address all statements. Please choose the appropriate response for each item:

	not correct at all					fully correct
I have been able to cope with daily assignments	0	1	2	3	4	5
I have felt internal fulfilment	0	1	2	3	4	5
I have felt comfortable	0	1	2	3	4	5
I have been able to enjoy life	0	1	2	3	4	5
I have been satisfied with my performance	0	1	2	3	4	5
I have been in agreement with my physical state	0	1	2	3	4	5
I have felt real pleasure	0	1	2	3	4	5

DSF, Frage 16, S.6, FW7 Herda, Scharfenstein u. Basler 1998

26b. The questions below address how you felt and how you were doing over the past 4 weeks.
Please tick off the statement that corresponds best to your condition.

During the past 4 weeks, how often have you been …

...very nervous?
O always O mostly O often O sometimes O rarely O never

...so down that nothing cheered you up?
O always O mostly O often O sometimes O rarely O never

...calm and composed?
O always O mostly O often O sometimes O rarely O never

...discouraged and sad?
O always O mostly O often O sometimes O rarely O never

...happy?
O always O mostly O often O sometimes O rarely O never

SF-36 Hogrefe Verlag, Bullinger u. Kirchberger 1998, Frage 9b, 9c, 9d, 9f und 9h

27. I know medical facilities that cater specifically for musicians' health problems
such as outpatient wards for musicians, institutes for musicians´ medicine, medical consultations for musicians etc.

O yes O no

If so, which? ____

27a. I have consulted such a facility

O no
O yes, because of pain
O yes, for some other reason:_________________________________

27b. Treatments I received there helped me significantly

O yes O no

Which treatments did you receive? _________________________________

28. (Apart from your pain) are you suffering from any other illnesses or effects of such?

This refers to diseases of various organ systems:

e.g. brain and spinal cord, nervous system, musculoskeletal system, connective tissue, cardiovascular system, thyroid, gastrointestinal problems, liver, gall bladder or pancreas, kidneys, lower urinary tract, metabolic or skin disorders, mental ailments,
other

O yes O no

If so, which? ____

29.Do you smoke?

O yes O no

30. How often do you have a drink containing alcohol?

O never O monthly or less O 2-4 times a month

O 2-3 times a week O 4 times or more a week

Audit-C©, Suchtforschungsverbund Baden-Württemberg, UKL Freiburg, Frage a

How many drinks containing alcohol do you have on a typical day when you are drinking?
(One drink containing alcohol means: 0,33l beer or 0,25l wine or 0,25l sparkling wine or 0,02l spirits)

O 1 or 2 O 3 or 4 O 5 or 6

O 7, 8 or 9 O 10 or more

Audit-C©, Suchtforschungsverbund Baden-Württemberg, UKL Freiburg, Frage b

How often do you have six or more drinks on one occasion?

O never O less than monthly O monthly

O weekly O more than weekly

Audit-C©, Suchtforschungsverbund Baden-Württemberg, UKL Freiburg, Frage c

31. Do you take other stimulants or narcotic substances?

O yes O no

which kind? ____

How often?

O never O less than monthly O monthly

O weekly O more than weekly

Workload and general degree of satisfaction

32. How often do you suffer from stage fright prior to a performance?

O every time O almost every time O sometimes O rarely O never

32a. How much does stage fright affect you?

Indicate the extent on the scale below:
0 means no stress, 10 means highest level of stress.

Stage fright 1 2 3 4 5 6 7 8 9 10

33. How often do you perceive the noise level at a concert or rehearsal as too loud?

O every time O almost every time O sometimes O rarely O never

33a. How much do you feel stressed by the high noise level in the orchestra?

High noise level	1	2	3	4	5	6	7	8	9	10

34. Please tick off the statement that corresponds to your condition in each line:

	not correct at all					fully correct
I am satisfied with my current occupation as a musician	0	1	2	3	4	5
I am worried about my professional future	0	1	2	3	4	5
I feel respected by my colleagues	0	1	2	3	4	5

35. Please tick off the statement that corresponds to your condition in each line:

	not correct at all					fully correct
I can talk to my colleagues about my health problems	0	1	2	3	4	5
I feel supported in my professional career by family and friends	0	1	2	3	4	5
My occupation creates problems with my partner/family	0	1	2	3	4	5

36. I generally feel I can meet...

	not correct at all					fully correct
... professional expectations	0	1	2	3	4	5
... private expectations	0	1	2	3	4	5
... social expectations	0	1	2	3	4	5
... my own expectations	0	1	2	3	4	5

... and a personal note at the end ...

Is there any question you consider very important in this context which we have not addressed?

____

Any other ideas or questions for us?

____
